# Cardioprotective effects of curcumin and carvacrol in doxorubicin‐treated rats: Stereological study

**DOI:** 10.1002/fsn3.1210

**Published:** 2019-09-10

**Authors:** Zahra Jafarinezhad, Ali Rafati, Farzaneh Ketabchi, Ali Noorafshan, Saied Karbalay‐Doust

**Affiliations:** ^1^ Histomorphometry and Stereology Research Center Shiraz University of Medical Sciences Shiraz Iran; ^2^ Department of Physiology Shiraz University of Medical Sciences Shiraz Iran; ^3^ Department of Anatomy Shiraz University of Medical Sciences Shiraz Iran

**Keywords:** carvacrol, curcumin, doxorubicin, stereology

## Abstract

Doxorubicin (DOX) is a cardiotoxic drug. To reduce the harmful effects of DOX, two plant‐derived components, including curcumin (CUR) and carvacrol (CAR), were considered. This study aimed to assess the protective effects of CUR and CAR on DOX‐induced cardiotoxicity using physiological and stereological evaluations. Male rats were randomly allocated to six groups. Group's I‐VI received phosphate‐buffered saline (PBS), CUR (100 mg kg^−1^ day^−1^), CAR (50 mg kg^−1^ day^−1^), DOX (4 mg kg^−1^ week^−1^), DOX‐CUR, and DOX‐CAR, respectively. On day 24, plasma troponin I and ECG were analyzed and the left ventricle underwent stereological assessment. The results showed a fivefold increase in troponin I in the DOX‐treated animals compared to the PBS ones. Additionally, heart rate and QRS amplitude, respectively, reduced by 18% and 31% and QT interval and QRS duration, respectively, increased by 41% and 24% in the DOX group in comparison with the PBS rats (*p* < .05). The total volume of the myocardium and vessels and the number of cardiomyocyte nuclei also, respectively, decreased by 30%, 45%, and 43% in the DOX group compared to the PBS animals (atrophy of the ventricular tissues, *p* < .01). Besides, the mean volumes of the connective tissue and cardiomyocytes, respectively, increased by 46% and 52% in the DOX group (*p* < .01). In the DOX‐CUR and DOX‐CAR groups, the changes were prevented extensively in comparison with the DOX group (*p* < .01). Co‐administration of CUR or CAR and doxorubicin for 24 days could improve the heart function and structural changes.

## 1 INTRODUCTION

The most frequent etiologies of heart failure include ischemic heart disease, hypertension, diabetes, and toxins (e.g., alcohol and cytotoxic drugs) (Kemp & Conte, 2012; Pontes et al., 2010). Along with these toxic agents, doxorubicin (DOX) was considered in the present study. It is one of the most effectual antitumor agents, but the clinical use of this drug has been seriously limited due to its toxicities (Chatterjee, Zhang, Honbo, & Karliner, 2010; El‐Sayed, Mansour, & Abdul‐Hameed, 2016; Octavia et al., 2012; Pontes et al., 2010; Takemura & Fujiwara, 2007). Up to now, many attempts have been made to soothe DOX side effects, including “dose optimization” and “combined therapy.” Recently, attempts have also been made to build up a combination therapy with medicinal plants to decrease the harmful properties of DOX (Singal, Li, Kumar, Danelisen, & Iliskovic, 2000). Thus, two components with plant origin, namely curcumin (CUR) and carvacrol (CAR), were evaluated in this study to minimize the cardiac side effects of DOX.

Curcumin is a yellow spice derived from the rhizome of curcuma longa linn. It is the active element of turmeric (Duan et al., 2012). It has been established that CUR has multiple pharmacological properties. Furthermore, CUR, as an agent for novel heart failure therapy, could inhibit the hypertrophic responses in cardiomyocytes (Katanasaka, Sunagawa, Hasegawa, & Morimoto, 2013). CAR is a major phenol compound, which can be found in various essential oils among plant species, such as origanum, thymus, and coridothymus. CAR has shown pharmacological abilities, as well (El‐Sayed et al., 2016; Yu, Liu, & Zhu, 2007). In the case of heart diseases, CAR has shown some effective properties (Chen et al., 2017; Yu et al., 2007). Therefore, two plant‐derived compounds, that is, CUR and CAR, were studied here in the case of cardiac toxicity.

Although many characteristics of DOX toxicity have been reported, quantitative aspects of the structural changes of DOX cardiotoxicity have received less attention. Therefore, the present research aims to answer the following questions using physiological, biochemical, and unbiased stereological methods. Do ECG and troponin I change after DOX treatment? How much do the volumes of the ventricle, myocardium, and connective tissue change after DOX treatment? How many cardiomyocyte nuclei would be lost/remained after cardiotoxicity induction via DOX? Would the mean volume of the cardiomyocytes change after cardiotoxicity induction? Would the length and volume of the microvessels of the ventricle change after DOX treatment? Are CUR and CAR effective in preventing changes in ECG and troponin I level after DOX exposure? Are CUR and CAR (solely or in a combined form) effective in preventing changes in the ventricular structure after DOX exposure?

## 2 MATERIALS AND METHODS

### 2.1 Animals and experimental design

In this study, 36 male adult Sprague Dawley rats weighing 220–250 g were obtained from the Experimental Animal Research Center of the University. All rats were housed under standard conditions (temperature: 22 ± 2°C, relative humidity: 50%, with a 12‐hr light/dark cycle) and had free access to laboratory food and water. The animals were subjected to an acclimatization period of 2 weeks before the beginning of the experiments. All procedures were approved (approval no. 95‐01‐01‐12152) by the Ethics Committee of the University. The rats were randomly allocated to six groups (*n* = 6). The phosphate‐buffered saline (PBS) group received PBS intraperitoneally on days 1, 8, 15, and 22. Groups CUR and CAR received 100 mg kg^−1^ day^−1^ CUR (Merck) and 50 mg kg^−1^ day^−1^ CAR (Sigma‐Aldrich) by oral gavages, respectively. Group DOX received 4 mg/kg DOX (Cell Pharm GmbH) through intraperitoneal injections on days 1, 8, 15, and 22 (Anjos Ferreira et al., 2007). The rats in the next two groups received DOX plus CUR and DOX plus CAR (Cetik, Ayhanci, & Sahinturk, 2012; Noorafshan, Asadi‐Golshan, Monjezi, & Karbalay‐Doust, 2014). The rats were sacrificed on day 24. The dose of DOX was chosen according to the previous reports. At this dose, DOX showed antineoplastic activity in addition to its cardiotoxic effect (Anjos Ferreira et al., 2007). The doses of CUR and CAR were also selected according to earlier results, revealing protective effects as well as lower side effects on other organs (Cetik et al., 2012). The rats’ body weights were measured on days 1, 8, 15, and 24. On day 24, the rats were anesthetized using 60 mg/kg sodium thiopental and ECG was recorded. Besides, at least 3 ml blood samples were obtained from the right atrium for troponin I assay. In doing so, the blood was allowed to clot. Then, the samples were centrifuged and the isolated sera were assayed.

### 2.2 Electrocardiogram evaluation

After anesthesia, the rats’ tongues were placed out of their mouths to avoid airway closure. During ECG recording, the animals were laid at supine position and kept warm to avert hypothermia. The animals were also located away from any metal objects to prevent noise. Additionally, their hands and feet were placed in a line. ECG was recorded through animal Bio Amp (FE136, ADInstruments) connected to the acquisition system (PowerLab, ADInstruments). ECG analysis was performed by Pro LabChart software. To record lead II, negative, positive, and earth pin electrodes were positioned on the right arm, left leg, and right leg, respectively. The ECG was recorded for about 3 min for each animal, and heart rate, QT interval, QRS duration, and QRS amplitude were evaluated (Parasuraman & Raveendran, 2012).

### 2.3 Troponin I assay

Plasma troponin I level is a specific indicator of damage to the myocardium. Cardiac troponin I (cTn‐I) was estimated using cardiac troponin ELISA kit (Cardinale et al., 2006; Reagan et al., 2013). The concentrations were obtained from the standard curve using serial dilutions (El‐Sayed et al., 2016).

### 2.4 Estimation of the ventricular volume and tissue shrinkage

The left ventricle was cleaned, and its total volume was estimated using the Scherle method. To determine the shrinkage and length density of microvessels, isotropic uniform random sections were required. These sections were obtained according to the “orientator method.” After tissue sectioning (4 and 25 µm thick) and staining (with hematoxylin, eosin, and Heidenhain's AZAN trichrome), the degree of tissue shrinkage “d (shr)” was calculated (Mühlfeld, Nyengaard, & Mayhew, 2010; Noorafshan, 2014; Nyengaard, 1999).

### 2.5 Estimation of the volume of the favored structures

The video microscopy system was used. The point‐counting method was applied on the systematically random sampled field on each histological slide. Finally, the volume density “*V*_V_ (structure/ref)” of the myocardium, vessels, and connective tissue was estimated using the following formula:

$$V_{v}\;\left(\text{structure/ref}\right)=P\;(\text{structure)}/P\left(\text{ref}\right)$$

where “P (structure)” and “P (ref)” represented the total number of the points hitting the favored structures and the ventricle sections, respectively. The total volumes of the myocardium, vessels, and connective tissue were estimated by multiplying *V*_V_ (structure/ref) by “V (ventricle)” (Mühlfeld et al., 2010; Noorafshan, 2014; Nyengaard, 1999).

### 2.6 Estimation of the numerical density of cardiomyocyte nuclei

The optical disector method was applied to estimate the numerical density using a 100× oil immersion objective lens with numerical aperture of 1.4 and a microcator. Afterward, an unbiased counting frame was superimposed on the 25‐µm‐thick tissue sections. The numerical density of cardiomyocyte nuclei in the ventricle, *N*_V_ (nuclei/ventricle), was obtained using the following formula:

$$N_{v}\left(\text{nuclei/ventricle}\right)=[\sum \bar{Q}/(\sum P.\;\text{(a/f)}.\;h)]\text{.}\;(t\text{/BA)}$$

where “∑*Q* ^−^” was the total number of the counted nuclei, “∑P” was the total number of points falling on the myocardium, “a/f” was the area of the counting frame (here 1,300 µm^2^), “*h*” was the height of the disector, “*t*” was the mean section thickness measured at different places of the section, and BA was the setting of the microtome for sectioning of the block. The “guard zones” and “*h*” were determined according to the z‐axis distribution of the nuclei (Mühlfeld et al., 2010; Noorafshan, 2014; Nyengaard, 1999).

### 2.7 Estimation of the mean cardiomyocyte volume

According to the “nucleator” method, the number‐weighted mean volume of the cells was estimated using a disector (number‐weighted). The following formula was applied:

$$V_{N(\text{cardiomyocyte})}=\frac{4\pi }{3}{\bar{l}}_{n}^{3}$$

where “${\bar{l}}_{n}^{3}$” was the average cubed intercept length across the cardiomyocyte through the center of the sampled nucleus (Mühlfeld et al., 2010; Noorafshan, 2014; Nyengaard, 1999).

### 2.8 Estimation of the microvessel length

The length density of the microvessels (≤10 µm) was estimated using the following formula:

LV(vessels/ventricle)=2∑Q/[∑P×(a/f)]

where “∑*Q*” denoted the total number of the vessel profiles counted per ventricle, “∑P” was the total number of the counted frames, and “a/f” was the area of the counting frame (Mühlfeld et al., 2010; Noorafshan, 2014; Nyengaard, 1999). The total unshrunken length was obtained by the following formula:

$${L\;(microvessels=LV\left(\text{vessels/ventricle}\right)\times [1-d\;\left(\text{shr}\right)}^{2/3}\times V\;\left(\text{ventricle}\right).$$

### 2.9 Statistical methods

Body weight and troponin were analyzed using *t* test for comparison of two groups and one‐way ANOVA for multiple comparisons. Other data were analyzed using Kruskal–Wallis and Mann–Whitney *U* test. *p* < .05 with adjusted alpha was considered to be statistically significant.

## 3 RESULTS

### 3.1 Weights

The results showed no significant differences among the animals’ body weights before different treatments. The animals’ weights increased by 12% in the PBS group over 24 days. In the DOX‐treated group, the animals had a 9% weight loss after 24 days of treatment. On the other hand, the DOX‐CUR and DOX‐CAR groups showed a lesser weight loss compared to the DOX‐treated rats (*p* < .05).

**Figure 1:**
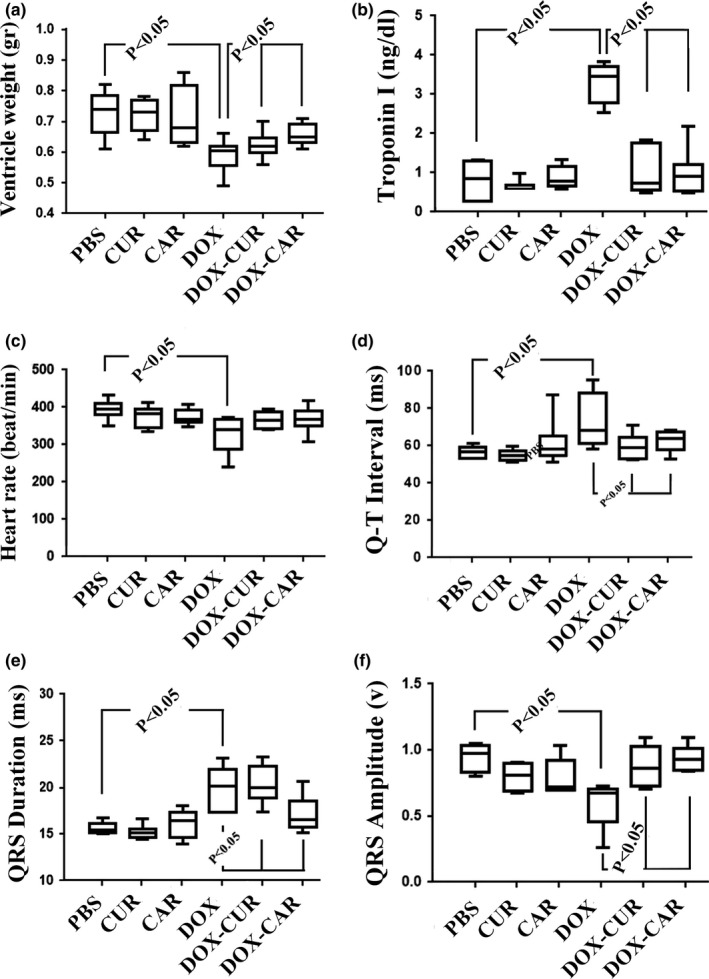
The box plots of the ventricle weight, troponin I level, and ECG parameters in different groups, including phosphate‐buffered saline (PBS), curcumin (CUR), carvacrol (CAR), doxorubicin (DOX), DOX‐CUR, and DOX‐CAR. (a) Left ventricle weight. (b) Troponin I level. (c) Heart rate (beats/min). (d) QT interval. (e) QRS duration. (f) QRS amplitude. The significant differences have been shown on the plot. *p* < .05, PBS versus DOX, DOX versus DOX‐CUR, and DOX versus DOX‐CAR

The ventricle weight reduced by 13% in the DOX‐treated group in comparison with the PBS group (*p* < .05). However, it showed a lower weight loss in the DOX‐CUR and DOX‐CAR groups compared to the DOX‐treated group (*p* < .05) (Figure 1).

There was no statistically significant difference between the DOX‐CUR and DOX‐CAR groups with respect to animal and ventricle weights.

### 3.2 Troponin I level

The results showed a fivefold increase in troponin I level in the DOX‐treated groups compared to the PBS group. However, troponin I levels were recovered in the DOX‐CUR and DOX‐CAR groups in comparison with the DOX group (*p* < .05) (Figure 1). There was no statistically significant difference between the DOX‐CUR and DOX‐CAR groups regarding troponin I level.

### 3.3 Electrocardiogram evaluation

The electrocardiogram findings have been presented in Figure 1. Accordingly, heart rate and QRS amplitude, respectively, reduced by 18% and 31% in the DOX‐treated group in comparison with the PBS group (*p* < .05). However, QT interval and QRS duration, respectively, increased by 41% and 24% in the DOX group in comparison with the PBS group (*p* < .05).

The results indicated no significant improvements in the heart rate of the DOX animals treated with CUR and CAR compared to the DOX group (*p* < .05). Yet, QRS amplitude, QT interval, and QRS duration were recovered in the DOX animals treated with CUR and CAR in comparison with the DOX group (*p* < .05) (Figure 1).

There was no statistically significant difference between the DOX‐CUR and DOX‐CAR groups concerning the aforementioned electrocardiogram findings.

### 3.4 Stereological evaluations

**Figure 2:**
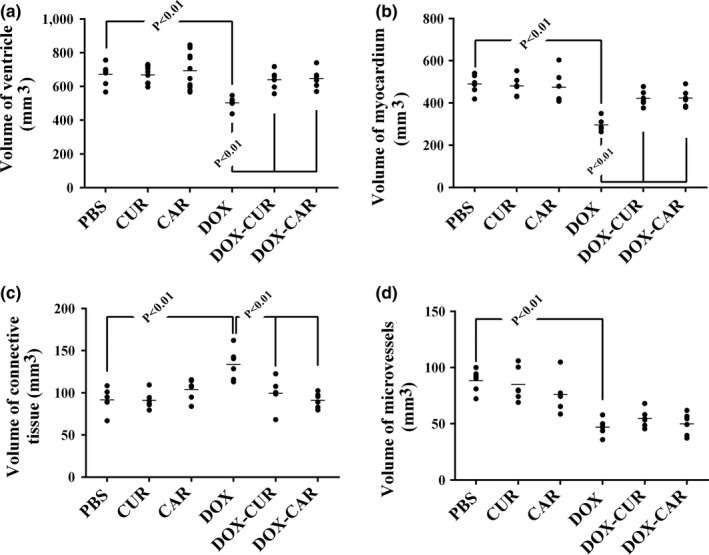
The scatter plots of the quantified parameters of the ventricle in different groups, including phosphate‐buffered saline (PBS), curcumin (CUR), carvacrol (CAR), doxorubicin (DOX), DOX‐CUR, and DOX‐CAR. The volumes of the ventricle (a), myocardium (b), connective tissue (c), and microvessels (d) have been presented. Each dot represents an animal, and the horizontal bars indicate the means of the parameters in the study groups. *p* < .01, PBS versus DOX, DOX versus DOX‐CUR, and DOX versus DOX‐CAR

**Figure 3:**
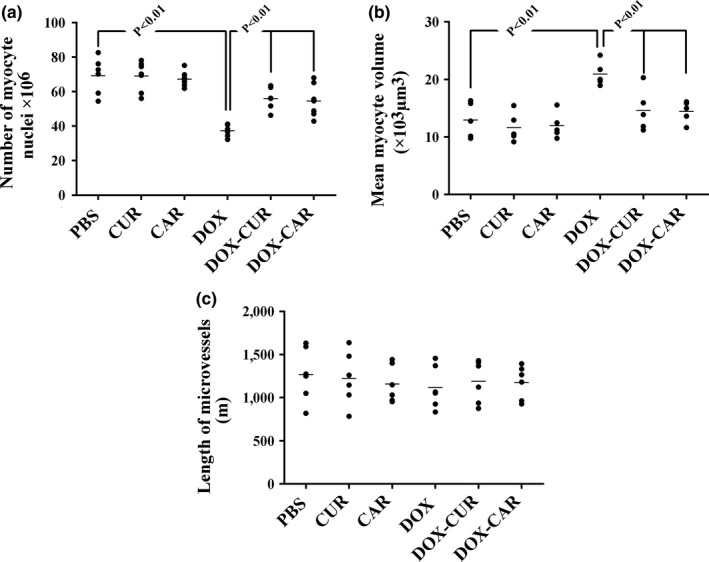
The scatter plots of the quantified parameters of the ventricle in different groups, including phosphate‐buffered saline (PBS), curcumin (CUR), carvacrol (CAR), doxorubicin (DOX), DOX‐CUR, and DOX‐CAR. The number of myocyte nuclei (a), mean volume of myocytes (b), and length of microvessels (c) have been presented. Each dot represents an animal, and the horizontal bars indicate the means of the parameters in the study groups. *p* < .01, PBS versus DOX, DOX versus DOX‐CUR, and DOX versus DOX‐CAR

The quantitative findings have been presented in Figures 2 and 3. Accordingly, the total volume of the myocardium and vessels and the number of cardiomyocyte nuclei, respectively, decreased by 30%, 45%, and 43% in the DOX‐treated group in comparison with the PBS‐treated animals (*p* < .01). However, the total volume of the connective tissue and the mean cardiomyocyte volume, respectively, increased by 46% and 52% in the DOX‐treated rats in comparison with the PBS‐treated animals (*p* < .01). These parameters changed in the DOX + CUR and DOX + CAR groups, but to a lesser extent, compared to the DOX‐treated group (*p* < .01). It should be noted that no statistically significant differences were found between the DOX‐CUR and DOX‐CAR groups with respect to the above‐mentioned parameters. Nonetheless, treatment of the animals with DOX (solely or combined with CUR or CAR) did not affect the length of the microvessels.

### 3.5 Qualitative changes

**Figure 4:**
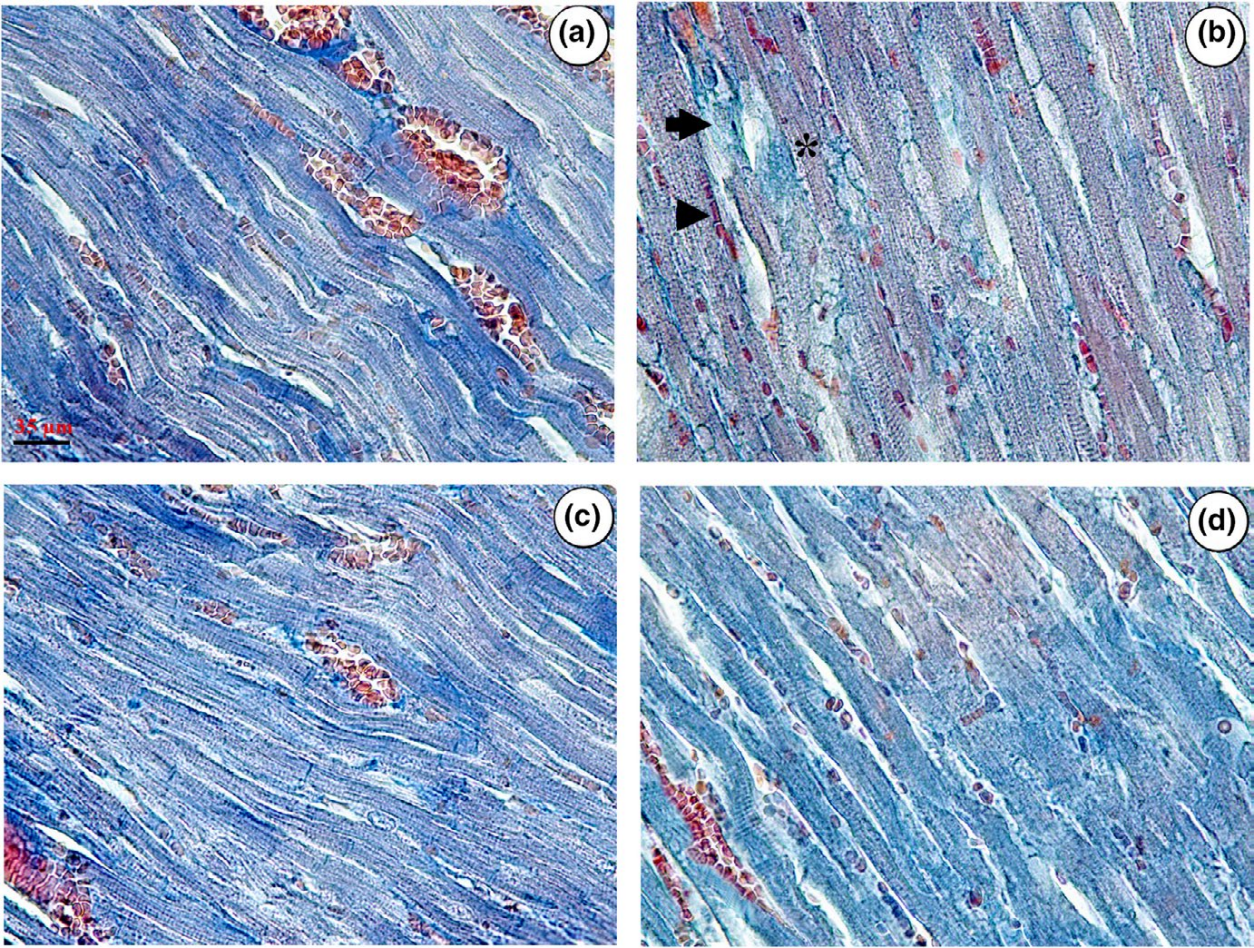
Microscopic evaluation of the ventricular tissue in different groups, including phosphate‐buffered saline (PBS) (a), doxorubicin (DOX) (b), DOX + CUR (c), and DOX + CAR (d). More connective tissues (arrow) and fewer muscles (asterisk) or vessel profiles (arrowhead) can be seen in the DOX‐treated ventricles. A recovery can be seen in the connective tissues, muscles, or vessels in the DOX‐CUR and DOX‐CAR groups

The photomicrographs of the ventricular structures in different groups have been presented in Figure 4. Briefly, the findings indicated the protective effects of CUR, CAR, or their combination on the atrophic changes of the myocardium, vessels, reduction in the cardiomyocyte population, formation of the fibrous tissue, and hypertrophy of the cardiomyocytes in the DOX‐treated rats.

## 4 DISCUSSION

The present study compared the possible protective effects of CUR and CAR on cardiac toxicity induced by DOX. The first step of the present study evaluated the effects of DOX on the left ventricle. The study results revealed weight loss in the DOX‐treated animals (Swamy, Gulliaya, Thippeswamy, Koti, & Manjula, 2012). Chang et al. (2015) examined the impact of 3 mg kg^−1^ week^−1^ DOX for 6 weeks and reported that weight increased until the 3rd week from baseline and then decreased from the 4th to the 9th week in all DOX‐only animals. In the present study, weight loss in the DOX group was seen from the first week, which continued throughout the study. However, lower weight loss was observed in the DOX‐CAR and DOX‐CUR groups. This indicates the protective effects of CUR and CAR in this case.

Other evaluated parameters were ventricle weight/volume. Here, unbiased stereological methods were applied to quantify the ventricular structure in details and to evaluate any adverse effects or improvements using comparable and reliable data. This method provides the quantitative data that show any changes in different conditions. In addition, these types of data provide the ground for determining the component of the ventricular structure that is more vulnerable to injury or has undergone improvement. To estimate the ventricle structure parameters, it is necessary to determine the reference volume, here the left ventricle, which is usually determined by the Cavalieri method. Cavalieri technique requires consecutive sectioning and high workload. However, reference volume was assessed using a combined method in the present study. Scherle method and shrinkage estimation on the isotropic uniform random sections were combined. The result is low workload without the need for consecutive sectioning. In fact, the analysis is performed on a few microscopic slides in this method (Noorafshan, 2014). The study results showed a reduction in the ventricle weight/volume, which could be correlated directly to the reduction of the myocardium volume and the cardiomyocyte population caused by DOX toxicity. The results also showed that the remained cardiomyocytes had undergone a hypertrophic growth as it was seen in the estimation of the mean volume of the cardiomyocytes. However, the hypertrophic growth was not enough to restore the changes in the weight/volume.

ECG recording showed a reduction in the heart rate and QRS amplitude and increment of the QT interval and QRS duration in the DOX‐treated rats. This is in agreement with the findings of the study by Shah, Mali, Zambare, and Bodhankar (2012), which showed increased ST, QT, and QRS complex in the rats treated with the same dose of DOX (4 mg/kg). The increment of serum troponin I level shortly after chemotherapy of patients with DOX is a strong predictor of ventricular dysfunction and poor cardiac outcome (Cardinale et al., 2006; Reagan et al., 2013). In an animal study, El‐Sayed et al. (2016) measured serum troponin I levels in DOX‐induced cardiotoxicity and showed its increment in toxic rats. Similar results were also obtained in the present study.

The second step of the present research evaluated the protective effects of CUR, CAR, and their combination on DOX cardiotoxicity. According to the results, treatment of DOX animals with CUR or CAR led to ameliorating of ECG recording, troponin I level, and ventricular structure, which is in accordance with other researches conducted on the issue. Imbaby, Ewais, Essawy, and Farag (2014) also reported that oral administration of CUR with the same dose used in the current study attenuated DOX cardiotoxicity and improved ECG parameters (Imbaby et al., 2014). Indeed, El‐Sayed et al. (2016) indicated that administration of CAR (25 mg/kg) for 14 days before DOX exposure ameliorated the heart function, troponin I level, and oxidative stress parameters. Swamy et al. (2012) also reported the cardioprotective effect of CUR on the loss of myocardial fibers in the DOX‐exposed animals. They stated that this protective effect could be attributed to the antioxidant activity of CUR. In the same line, Mohamad et al. (2009) demonstrated the ameliorative effect of CUR against DOX‐induced cardiac toxicity in rats.

The protective effects of CUR and CAR in the present study could be attributed to the anti‐inflammatory, anti‐apoptosis, and antioxidant properties of these components. It has been confirmed that CUR and CAR possessed antioxidant activities, which could result in a reduction in lipid peroxidation and apoptosis (Duan et al., 2012; El‐Sayed et al., 2016).

## 5 CONCLUSIONS

In conclusion, DOX treatment induced changes in troponin I level and ECG parameters. In addition, atrophic changes of the myocardium and vessels, reduction of cardiomyocyte nuclei, and increment of connective tissue could be seen after 24 days of DOX treatment. Yet, CUR (100 mg kg^−1^ day^−1^) and CAR (50 mg kg^−1^ day^−1^) could protect DOX cardiotoxicity.

## CONFLICT OF INTEREST

The authors declare that they do not have any conflict of interest.

## ETHICAL APPROVAL

This study was approved by the Animal Care and Ethics Committee of the University (agreement license: 95‐01‐01‐12152).

## INFORMED CONSENT

Written informed consent was obtained from all study participants.
